# Supplementary material for: Mitochondrial Kv1.3 Channels as Target for Treatment of Multiple Myeloma
Source: Cancers (Basel). 2022 Apr 13;14(8):1955. doi: 10.3390/cancers14081955 (PMC9032553; doi:10.3390/cancers14081955)
Supplement: Supplementary file 1 [file cancers-14-01955-s001.zip › cancers-1634161-File S1.pdf]

Blot from Figure 1 a

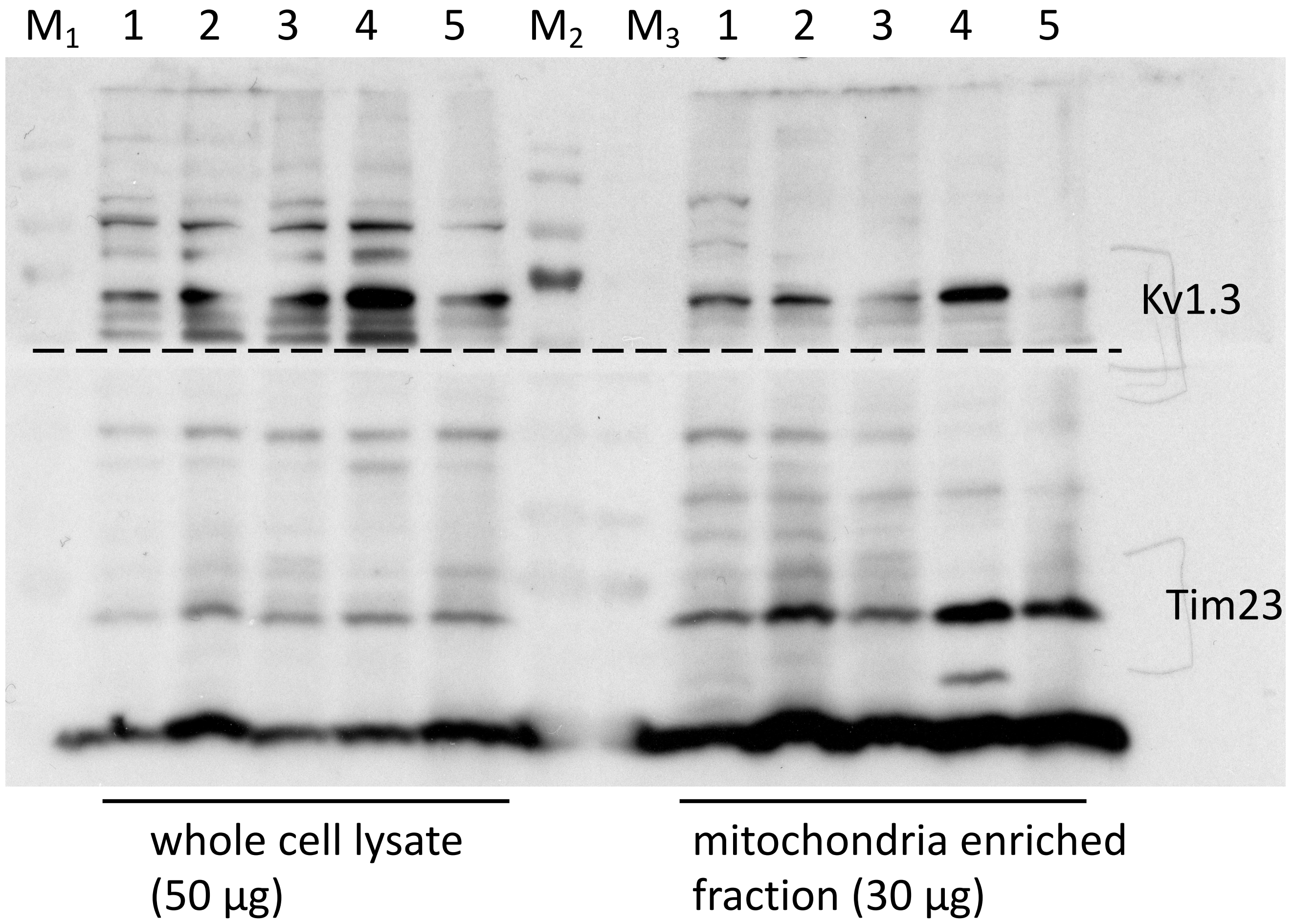

$M_{1,3}$ = Marker (2.5 µl)
1 = U266
2 = L-363
3 = RPMI-8226
4 = Jurkat
5 = MOPC
M_2 = Marker (5 µl)

Gel: 8.5%

Marker = Thermo Scientific PageRuler Prestained Protein Ladder #26616
