# Supplementary material for: Mitochondrial Kv1.3 Channels as Target for Treatment of Multiple Myeloma
Source: Cancers (Basel). 2022 Apr 13;14(8):1955. doi: 10.3390/cancers14081955 (PMC9032553; doi:10.3390/cancers14081955)
Supplement: Supplementary file 1 [file cancers-14-01955-s001.zip › cancers-1634161-supplementary.pdf]

Supplementary Table S1. EC50 values for PAPTP and PCARBTP in patient-derived multiple myeloma cells. EC50 values for PAPTP and PCARBTP were calculated for each patient derived plasma-cell clone.

1st diagnosis standard risk cytogenetics		
**Patient**	**EC50 [μM] PAPTP**	**EC50 [μM] PCARBTP**
# 15	< 0.001	<0.001
# 12	0.03	1.04
# 23	0.03	0.06
# 19	0.25	0.62
# 27	0.28	0.68
# 5	0.46	1.16
# 26	0.47	1.18
# 13	0.53	1.38
# 16	0.58	2.27
# 7	0.59	2.05
# 21	0.64	1.6
# 18	0.68	1.7
# 24	2.20	5.53
# 8	Non-responsive	Non-responsive
**1st diagnosis high-risk cytogenetics**		
# 4	0.02	0.6
# 11	0.03	1.08
# 25	0.03	0.06
# 1	0.32	2.27
# 17	1.30	4.43
# 9	4.80	Non-responsive
**Relapsed/refractory**		
# 10	0.02	0.15
# 14	0.13	0.002
# 3	0.55	1.40
# 2	0.56	1.40
# 20	0.70	2.35
# 22	3.13	7.82
# 6	3.36	3.50

Supplementary Table S2. Up-regulated genes in RPMI8226-R1.0 and L363-R3.5. In RPMI8226-R1.0 cells 74 genes have been up-regulated > 2 log2-fold (p-value < 0.01) and 7 genes ⩾ 2-fold (adjusted p-value < 0.01) that have been up-regulated in L363-R3.5 in comparison to the respective parental cell line. Only one gene ABCB1 (MDR-1; highlighted in orange)is up-regulated in both RPMI8226-R1.0 and L363-R3.5 cells.

RPMI8226			
ID	log2-fold change	p-value (adj.)	Gene
ENSG00000180777	5,19	1,3952E-42	ANKRD30B
ENSG00000145242	4,76	2,5409E-28	EPHA5
ENSG00000147655	4,73	5,5233E-24	RSPO2
ENSG00000154975	4,36	1,4681E-19	CA10
ENSG00000180828	4,35	1,5426E-19	BHLHE22
ENSG00000169439	4,26	1,801E-67	SDC2
ENSG00000165995	4,19	7,6445E-19	CACNB2
ENSG00000085563	4,01	1,7459E-21	ABCB1
ENSG00000183206	3,80	4,0518E-14	POTEC
ENSG00000008517	3,74	2,8474E-89	IL32
ENSG00000114861	3,58	5,9767E-34	FOXP1
ENSG00000173114	3,48	4,9711E-13	LRRN3
ENSG00000137766	3,44	1,8174E-82	UNC13C
ENSG00000175302	3,30	3,5336E-10	ANKRD30BP1
ENSG00000234435	3,21	1,0164E-48	LINC01432
ENSG00000136732	3,18	1,1936E-29	GYPC
ENSG00000102924	3,18	1,6803E-15	CBLN1
ENSG00000198695	3,12	1,401E-162	MT-ND6
ENSG00000148513	3,12	6,4282E-10	ANKRD30A
ENSG00000086967	3,11	1,1194E-19	MYBPC2
ENSG00000145850	3,11	2,022E-15	TIMD4
ENSG00000158639	3,06	1,1574E-26	PAGE5
ENSG00000162745	3,05	1,9565E-13	OLFML2B
ENSG00000111913	3,00	3,7939E-09	RIPOR2
ENSG00000152092	2,93	6,7811E-08	ASTN1
ENSG00000152767	2,91	3,3063E-19	FARP1
ENSG00000069188	2,88	5,0609E-12	SDK2
ENSG00000105784	2,83	3,0825E-08	RUNDC3B
ENSG00000133104	2,82	4,1605E-13	SPART
ENSG00000139926	2,81	3,2008E-08	FRMD6
ENSG00000144891	2,77	4,5906E-07	AGTR1
ENSG00000112419	2,76	1,2766E-07	PHACTR2
ENSG00000151150	2,75	5,6099E-08	ANK3
ENSG00000135643	2,74	6,8385E-13	KCNMB4
ENSG00000166025	2,67	1,9258E-07	AMOTL1
ENSG00000090104	2,64	2,6037E-09	RGS1
ENSG00000109971	2,59	2,8057E-73	HSPA8
ENSG00000171617	2,55	2,9846E-12	ENC1
ENSG00000080824	2,53	8,963E-124	HSP90AA1
ENSG00000083857	2,53	6,2935E-06	FAT1
ENSG00000205213	2,50	8,2371E-06	LGR4
ENSG00000182010	2,48	1,125E-09	RTKN2
ENSG00000120694	2,42	1,3673E-89	HSPH1
ENSG00000183454	2,41	2,8936E-06	GRIN2A
ENSG00000119139	2,39	6,737E-09	TJP2
ENSG00000186517	2,39	2,0446E-30	ARHGAP30
ENSG00000204388	2,35	5,1873E-21	HSPA1B
ENSG00000138735	2,34	2,9992E-19	PDE5A
ENSG00000252481	2,33	3,5371E-07	SCARNA13
ENSG00000198797	2,33	4,1589E-05	BRINP2
ENSG00000131711	2,32	1,1589E-08	MAP1B
ENSG00000172478	2,32	2,3809E-09	C2orf54
ENSG00000124191	2,31	8,6312E-35	TOX2
ENSG00000251381	2,30	4,4373E-07	LINC00958
ENSG00000196586	2,30	9,4349E-78	MYO6
ENSG00000160191	2,29	8,8754E-06	PDE9A
ENSG00000105976	2,28	8,3378E-09	MET
ENSG00000168952	2,25	1,6526E-05	STXBP6
ENSG00000275830	2,25	1,8938E-13	AL355974.2
ENSG00000210140	2,13	7,9093E-13	MT-TC
ENSG00000189127	2,10	0,000269	ANKRD34B
ENSG00000042286	2,09	1,7484E-09	AIFM2
ENSG00000148468	2,09	7,1565E-22	FAM171A1
ENSG00000140563	2,09	1,4769E-05	MCTP2
ENSG00000136634	2,08	0,00020053	IL10
ENSG00000163435	2,08	3,4488E-08	ELF3
ENSG00000122188	2,07	0,00027259	LAX1
ENSG00000140859	2,04	3,3004E-09	KIFC3
ENSG00000210194	2,04	1,9079E-05	MT-TE
ENSG00000061676	2,03	0,00044157	NCKAP1
ENSG00000226025	2,01	1,7007E-10	AC005515.1
ENSG00000198133	2,01	1,2103E-05	TMEM229B
ENSG00000100591	2,00	3,8716E-50	AHSA1
ENSG00000156127	2,00	2,5003E-06	BATF

L363			
ID	log2-fold change	p-value (adj.)	Gene
ENSG00000085563	5,12	2,06E-55	ABCB1
ENSG00000176049	2,79	2,27E-14	JAKMIP2
ENSG00000169550	2,72	1,40E-14	MUC15
ENSG00000107201	2,62	1,62E-12	DDX58
ENSG00000173068	2,57	6,26E-11	BNC2
ENSG00000170775	2,13	3,61E-09	GPR37
ENSG00000112320	2,09	1,12E-07	SOBP

Supplementary Table S3. Down-regulated genes in RPMI8226-R1.0 and L363-R3.5. In RPMI8226-R1.0 cells we have 183 genes that have been down-regulated > -2 log2-fold (p-value < 0.01) and 43 genes > -2 log2-fold (adjusted p-value < 0.01) have been down-regulated genes in L363-R3.5 in comparison to the respective parental cell line.

RPMI8226			
ID	log2-fold change	p-value (adj.)	Gene
ENSG00000156738	-5,14	1,91E-37	MS4A1
ENSG00000137959	-4,34	2,59E-18	IFI44L
ENSG00000132274	-4,11	1,04E-16	TRIM22
ENSG00000239998	-4,09	8,15E-17	LILRA2
ENSG00000172183	-4,04	1,88E-59	ISG20
ENSG00000186340	-4,01	4,14E-16	THBS2
ENSG00000231196	-3,92	1,18E-22	RP11-495P10.8
ENSG00000137965	-3,89	8,54E-29	IFI44
ENSG00000091436	-3,73	8,15E-17	MAP3K20
ENSG00000132185	-3,71	3,88E-106	FCRLA
ENSG00000172465	-3,69	8,53E-14	TCEAL1
ENSG00000137628	-3,68	3,97E-13	DDX60
ENSG00000155961	-3,63	1,80E-23	RAB39B
ENSG00000166750	-3,59	1,19E-23	SLFN5
ENSG00000135925	-3,57	3,11E-15	WNT10A
ENSG00000258732	-3,33	8,18E-12	AC025884.1
ENSG00000230615	-3,31	3,22E-20	AL139220.2
ENSG00000139269	-3,30	4,76E-52	INHBE
ENSG00000188282	-3,26	3,59E-10	RUFY4
ENSG00000267871	-3,25	1,16E-10	ZNF460-AS1
ENSG00000133169	-3,24	2,30E-21	BEX1
ENSG00000183971	-3,14	8,95E-13	NPW
ENSG00000123329	-3,13	2,74E-13	ARHGAP9
ENSG00000135046	-3,11	1,19E-27	ANXA1
ENSG00000141447	-3,11	1,45E-11	OSBPL1A
ENSG00000078081	-3,11	3,93E-13	LAMP3
ENSG00000204287	-3,08	1,42E-47	HLA-DRA
ENSG00000012223	-3,07	1,04E-16	LTF
ENSG00000132530	-3,07	5,51E-17	XAF1
ENSG00000233864	-3,07	5,16E-09	TTTY15
ENSG00000183287	-3,06	1,90E-10	CCBE1
ENSG00000198542	-3,02	1,62E-12	ITGBL1
ENSG00000104413	-3,01	1,52E-40	ESRP1
ENSG00000182667	-2,99	4,70E-10	NTM
ENSG00000181381	-2,94	2,79E-09	DDX60L
ENSG00000223865	-2,92	1,57E-13	HLA-DPB1
ENSG00000198521	-2,89	8,52E-09	ZNF43
ENSG00000184304	-2,89	6,48E-10	PRKD1
ENSG00000132846	-2,88	4,72E-21	ZBED3
ENSG00000196126	-2,86	5,29E-23	HLA-DRB1
ENSG00000142765	-2,86	1,84E-32	SYTL1
ENSG00000179241	-2,84	1,11E-10	LDLRAD3
ENSG00000169855	-2,84	3,34E-09	ROBO1
ENSG00000117228	-2,84	1,82E-14	GBP1
ENSG00000132141	-2,82	4,64E-13	CCT6B
ENSG00000163683	-2,81	9,36E-13	SMIM14
ENSG00000187720	-2,81	1,06E-07	THSD4
ENSG00000256568	-2,80	3,28E-07	AP002761.2
ENSG00000277443	-2,79	4,76E-68	MARCKS
ENSG00000110446	-2,78	4,63E-14	SLC15A3

L363			
ID	log2-fold change	p-value (adj.)	Gene
ENSG00000046774	-3,73	5,97E-26	MAGEC2
ENSG00000176406	-3,45	3,20E-19	RIMS2
ENSG00000007237	-3,21	1,75E-16	GAS7
ENSG00000124762	-3,19	3,07E-18	CDKN1A
ENSG00000107282	-3,07	1,20E-14	APBA1
ENSG00000180964	-3,05	1,20E-14	TCEAL8
ENSG00000007312	-3,03	1,75E-16	CD79B
ENSG00000111885	-2,96	1,50E-19	MAN1A1
ENSG00000232001	-2,92	3,11E-13	AC108868.2
ENSG00000028277	-2,78	5,40E-17	POU2F2
ENSG00000035862	-2,76	4,03E-16	TIMP2
ENSG00000008283	-2,69	5,96E-11	CYB561
ENSG00000163191	-2,69	9,21E-17	S100A11
ENSG00000154096	-2,66	1,04E-10	THY1
ENSG00000137766	-2,65	6,24E-12	UNC13C
ENSG00000155657	-2,63	9,13E-16	TTN
ENSG00000197471	-2,62	1,05E-16	SPN
ENSG00000143603	-2,57	4,40E-10	KCNN3
ENSG00000069188	-2,49	2,61E-09	SDK2
ENSG00000131171	-2,49	2,03E-09	SH3BGRL
ENSG00000131378	-2,39	3,65E-09	RFTN1
ENSG00000143119	-2,35	1,43E-11	CD53
ENSG00000272620	-2,32	3,10E-08	AFAP1-AS1
ENSG00000136026	-2,30	1,45E-12	CKAP4
ENSG00000162909	-2,29	3,93E-08	CAPN2
ENSG00000179542	-2,25	1,83E-09	SLITRK4
ENSG00000145703	-2,24	3,78E-19	IQGAP2
ENSG00000028137	-2,23	8,08E-08	TNFRS1B
ENSG00000143554	-2,18	1,04E-08	SLC27A3
ENSG00000154127	-2,16	2,86E-07	UBASH3B
ENSG00000122986	-2,14	1,20E-08	HVCN1
ENSG00000006042	-2,13	1,00E-06	TMTM98
ENSG00000197747	-2,12	6,70E-10	S100A10
ENSG00000170627	-2,11	1,44E-06	GTSF1
ENSG00000184060	-2,10	6,90E-09	ADAP2
ENSG00000184640	-2,09	1,62E-06	SEPTIN9
ENSG00000166681	-2,08	4,25E-09	BEX3
ENSG00000009790	-2,08	1,31E-09	TRAF3IP3
ENSG00000155926	-2,05	1,08E-09	SLA
ENSG00000172578	-2,05	1,13E-06	KLHL6
ENSG00000145685	-2,04	1,25E-06	LHFPL2
ENSG00000136167	-2,02	1,72E-11	LCP1
ENSG00000169116	-2,00	2,07E-06	PARM1

ENSG00000103056	-2,77	5,59E-10	SMPD3
ENSG00000227591	-2,75	8,95E-21	AL031316.1
ENSG00000169213	-2,72	5,20E-09	RAB3B
ENSG00000100968	-2,72	4,15E-10	NFATC4
ENSG00000157601	-2,71	1,94E-09	MX1
ENSG00000196664	-2,70	1,17E-11	TLR7
ENSG00000167800	-2,69	4,58E-07	TBX10
ENSG00000196735	-2,69	4,53E-07	HLA-DQA1
ENSG00000239039	-2,68	1,24E-14	SNORD13
ENSG00000128165	-2,66	1,34E-67	ADM2
ENSG00000183690	-2,66	1,48E-06	EFHC2
ENSG00000164627	-2,66	6,95E-07	KIF6
ENSG00000138755	-2,65	7,25E-07	CXCL9
ENSG00000116701	-2,64	2,06E-34	NCF2
ENSG00000115956	-2,64	4,34E-78	PLEK
ENSG00000116761	-2,64	4,24E-17	CTH
ENSG00000185507	-2,63	7,21E-19	IRF7
ENSG00000115414	-2,59	5,22E-72	FN1
ENSG00000157514	-2,57	1,75E-48	TSC22D3
ENSG00000163285	-2,56	4,79E-07	GABRG1
ENSG00000104972	-2,56	1,53E-07	LILRB1
ENSG00000174600	-2,55	2,88E-06	CMKLR1
ENSG00000167995	-2,53	6,13E-06	BEST1
ENSG00000164742	-2,52	3,18E-34	ADCY1
ENSG00000204936	-2,52	3,78E-14	CD177
ENSG00000114374	-2,52	2,91E-06	USP9Y
ENSG00000168016	-2,52	2,37E-14	TRANK1
ENSG00000163219	-2,50	1,44E-06	ARHGAP25
ENSG00000248498	-2,49	5,07E-06	ASNSP1
ENSG00000112799	-2,49	1,22E-32	LY86
ENSG00000122420	-2,48	7,78E-06	PTGFR
ENSG00000183486	-2,48	4,54E-06	MX2
ENSG00000137745	-2,46	1,22E-23	MMP13
ENSG00000126709	-2,46	6,54E-08	IFI6
ENSG00000231389	-2,46	3,71E-13	HLA-DPA1
ENSG00000138646	-2,45	1,71E-08	HERC5
ENSG00000229391	-2,45	2,65E-06	HLA-DRB6
ENSG00000102524	-2,45	1,70E-08	TNFSF13B
ENSG00000014257	-2,43	8,37E-19	ACPP
ENSG00000174080	-2,43	8,95E-07	CTSF
ENSG00000169896	-2,43	1,91E-06	ITGAM
ENSG00000170017	-2,43	2,50E-09	ALCAM
ENSG00000116117	-2,42	9,97E-07	PARD3B
ENSG00000175197	-2,42	5,25E-24	DDIT3
ENSG00000198692	-2,41	6,28E-21	EIF1AY
ENSG00000177337	-2,39	2,44E-09	DLGAP1-AS1
ENSG00000234936	-2,37	2,43E-08	AC010883.1
ENSG00000170909	-2,36	1,03E-05	OSCAR
ENSG00000172361	-2,35	7,08E-08	CFAP53
ENSG00000187608	-2,35	2,71E-06	ISG15
ENSG00000168961	-2,35	6,83E-08	LGALS9
ENSG00000130766	-2,34	5,89E-79	SESN2
ENSG00000151012	-2,33	3,04E-47	SLC7A11
ENSG00000265778	-2,33	1,61E-07	AC018413.1
ENSG00000168081	-2,33	7,11E-10	PNOC
ENSG00000263004	-2,32	1,10E-11	AC007114.1
ENSG00000019582	-2,32	7,08E-77	CD74
ENSG00000103546	-2,32	4,45E-05	SLC6A2
ENSG00000100889	-2,32	2,50E-32	PCK2
ENSG00000182568	-2,32	4,56E-10	SATB1
ENSG00000135218	-2,31	5,21E-06	CD36
ENSG00000235770	-2,31	1,63E-05	LINC00607
ENSG00000138835	-2,31	3,22E-07	RGS3
ENSG00000186832	-2,30	4,59E-05	KRT16
ENSG00000141682	-2,30	1,95E-38	PMAIP1
ENSG00000101188	-2,30	3,60E-05	NTSR1
ENSG00000162407	-2,29	3,77E-06	PLPP3
ENSG00000196517	-2,29	2,43E-26	SLC6A9
ENSG00000152926	-2,28	9,15E-06	ZNF117
ENSG00000185745	-2,28	1,22E-05	IFIT1
ENSG00000163220	-2,26	2,44E-18	S100A9
ENSG00000164647	-2,26	1,47E-07	STEAP1
ENSG00000167103	-2,25	4,50E-31	PIP5KL1
ENSG00000159239	-2,25	3,07E-12	AC005041.1
ENSG00000253276	-2,24	7,47E-13	CCDC71L
ENSG00000171791	-2,23	2,51E-47	BCL2
ENSG00000276231	-2,23	8,58E-05	PIK3R6
ENSG00000263412	-2,23	1,02E-06	AC004477.1
ENSG00000170345	-2,22	9,69E-05	FOS
ENSG00000129990	-2,22	1,09E-10	SYT5
ENSG00000087074	-2,21	2,87E-18	PPP1R15A
ENSG00000119917	-2,21	1,20E-07	IFIT3
ENSG00000131002	-2,21	2,02E-10	TXLNGY
ENSG00000074621	-2,20	5,79E-06	SLC24A1
ENSG00000163347	-2,19	4,75E-05	CLDN1
ENSG00000166927	-2,18	0,00010585	MS4A7
ENSG00000184939	-2,17	7,34E-12	ZFP90

ENSG00000164440	-2,15	6,41E-06	TXLNB
ENSG00000198496	-2,15	5,73E-09	NBR2
ENSG00000261652	-2,15	8,61E-10	C15orf65
ENSG00000263711	-2,15	7,92E-13	AC079062.1
ENSG00000226751	-2,15	5,13E-05	AF127936.1
ENSG00000144366	-2,14	2,29E-17	GULP1
ENSG00000233230	-2,14	6,65E-05	AC079807.1
ENSG00000170006	-2,12	8,41E-07	TMEM154
ENSG00000273906	-2,12	0,00022433	AC011297.1
ENSG00000184545	-2,12	3,24E-06	DUSP8
ENSG00000010219	-2,12	5,75E-07	DYRK4
ENSG00000182749	-2,10	0,00021277	PAQR7
ENSG00000129824	-2,09	5,60E-30	RPS4Y1
ENSG00000138642	-2,08	6,20E-05	HERC6
ENSG00000086730	-2,08	2,13E-14	LAT2
ENSG00000148175	-2,08	3,22E-51	STOM
ENSG00000105048	-2,08	3,93E-12	TNNT1
ENSG00000213462	-2,08	3,13E-08	ERV3-1
ENSG00000196668	-2,08	2,56E-18	LINC00173
ENSG00000133808	-2,07	2,46E-05	MICALC
ENSG00000155980	-2,07	0,00034075	KIF5A
ENSG00000172159	-2,06	2,66E-25	FRMD3
ENSG00000235961	-2,06	0,00021528	PNMA6A
ENSG00000115415	-2,05	1,03E-08	STAT1
ENSG00000092607	-2,05	0,00039139	TBX15
ENSG00000172264	-2,05	1,01E-10	MACROD2
ENSG00000144481	-2,05	1,82E-08	TRPM8
ENSG00000111058	-2,04	0,00029605	ACSS3
ENSG00000071991	-2,04	0,00032681	CDH19
ENSG00000244485	-2,04	7,14E-06	RPL18P13
ENSG00000188177	-2,04	3,01E-18	ZC3H6
ENSG00000181722	-2,04	3,89E-05	ZBTB20
ENSG00000149781	-2,03	5,22E-07	FERMT3
ENSG00000111335	-2,03	3,52E-08	OAS2
ENSG00000178662	-2,03	0,00041594	CSRNP3
ENSG00000206052	-2,02	1,33E-14	DOK6
ENSG00000164088	-2,02	6,15E-23	PPM1M
ENSG00000151632	-2,02	0,00049697	AKR1C2
ENSG00000111331	-2,01	7,38E-06	OAS3
ENSG00000233912	-2,01	9,82E-06	AC026202.2
ENSG00000151470	-2,00	6,13E-07	C4orf33
ENSG00000135116	-2,00	1,68E-06	HRK
ENSG00000198816	-2,00	0,00026937	ZNF358
ENSG00000108771	-2,00	5,72E-05	DHX58
ENSG00000185615	-2,00	2,57E-24	PDIA2
ENSG00000163218	-2,00	0,00055157	PGLYRP4
